# Draft Genome Sequence of *Mycobacterium tuberculosis* Strain MT11, Which Represents a New Lineage

**DOI:** 10.1128/genomeA.00573-15

**Published:** 2015-06-04

**Authors:** Djaltou Aboubaker Osman, Michael Phelippeau, Didier Musso, Catherine Robert, Caroline Michelle, Olivier Croce, Michel Drancourt

**Affiliations:** a Aix Marseille Université, URMITE, UMR CNRS 7278, IRD 198, INSERM 1095, Marseille, France; b Institut de Recherche Médicinale (IRM), Centre d’Études et de Recherche de Djibouti (CERD), Djibouti, Djibouti; c Institut Louis Malardé, Papeete, French Polynesia

## Abstract

We sequenced the genome of *Mycobacterium tuberculosis* strain MT11, which exhibits a specific 16S rRNA gene mutation found in 6% of French Polynesian *M. tuberculosis* isolates. It comprises a 4,110,293-bp chromosome with 65.15% G+C content, and it encodes 3,949 proteins and contains 85 predicted RNA genes. The TbD1 region is absent in strain MT11 as in modern *M. tuberculosis* strains.

## GENOME ANNOUNCEMENT

Pulmonary tuberculosis remains one of most deadly infectious diseases (1). The responsible agent, *Mycobacterium tuberculosis*, is a genetically monomorphic species, owing to its low DNA diversity (2) and clonal evolution (3), but with a host adaptation characterized by specific clones in some geographic areas (4–6). Among 34 *M. tuberculosis* isolates obtained from French Polynesian patients (7), we recently observed two isolates, MT11 and MT14, the peptidic spectra of which were clustered by matrix-assisted laser desorption ionization–time of flight mass spectrometry. Of note, the 16S rRNA gene sequences of both isolates showed 99% sequence similarity to that of *M. tuberculosis* H37Rv (GenBank accession no. AL123456) and a unique mutation at position 1247. This mutation differed from all 16S rRNA genes previously reported in the other *M. tuberculosis* complex members. The partial *rpoB* gene sequences of MT11 and MT14 showed 100% similarity with that of *M. tuberculosis* H37Rv (GenBank accession no. AL123456) (8). Twenty-four mycobacterial interspersed repetitive-unit–variable-number tandem-repeat (MIRU-VNTR) genotyping loci (9) and spoligotyping (10) identified the two isolates as belonging to the Haarlem lineage (O. D. Aboubaker, M. Phelippeau, M. Drancourt, and D. Musso, unpublished data). We thought that analyzing the whole-genome sequence of *M. tuberculosis* MT11 would help define the phylogenetic relationships within the *M. tuberculosis* complex and design tools for its advanced detection and identification.

Chromosomal DNA was isolated as previously described (11) and sequenced on the MiSeq Technology (Illumina, Inc., San Diego, CA, USA) through four runs using two mate-pair libraries with insert sizes of 10 and 3.3 kb in a 2 × 250-bp run for each barcoded library. The whole set of reads was trimmed using Trimmomatic (12) and assembled using the assembler software SPAdes (13, 14). Contigs were combined using SSPACE (15) and Opera (16), helped by GapFiller (17), and homemade tools in Python were used to refine the set.

The draft genome of *M. tuberculosis* MT11 consists of 16 contigs without gap containing 4,110,293 bp, which is the second smallest genome among *M. tuberculosis*, and a 65.15% G+C content. Noncoding genes and miscellaneous features were predicted using RNAmmer (18), Aragorn (19), Rfam (20), Pfam (21), and Infernal (22). Coding DNA sequences (CDSs) were predicted using Prodigal (23), and functional annotation was achieved using BLAST+ (24) and HMMER3 (25) against the UniProtKB database (26). The genome was shown to contain at least 85 predicted RNAs, including 3 rRNAs, 51 tRNAs, one transfer-messenger RNA (tmRNA), and 30 miscellaneous RNAs. A total of 3,949 genes were also identified, representing a coding capacity of 3,682,833 bp (89.6% coding density). Among these genes, 281 (7.12%) encode putative proteins, and 542 (13.72%) encode hypothetical proteins. Moreover, 2,776 genes matched a least one sequence in the Clusters of Orthologous Groups (COGs) database (27, 28) using BLASTp default parameters.

### Nucleotide sequence accession numbers. 

The *M. tuberculosis* MT11 strain annotated genome sequence has been deposited at EMBL under the accession numbers CVMX01000001 to CVMX01000016.
